# Machine Learning Models for Nocturnal Hypoglycemia Prediction in Hospitalized Patients with Type 1 Diabetes

**DOI:** 10.3390/jpm12081262

**Published:** 2022-07-31

**Authors:** Vladimir B. Berikov, Olga A. Kutnenko, Julia F. Semenova, Vadim V. Klimontov

**Affiliations:** 1 Laboratory of Endocrinology, Research Institute of Clinical and Experimental Lymphology—Branch of the Institute of Cytology and Genetics, Siberian Branch of Russian Academy of Sciences (RICEL—Branch of IC&G SB RAS), 630060 Novosibirsk, Russia; berikov@math.nsc.ru (V.B.B.); eekmxtyjr@yandex.ru (J.F.S.); 2 Laboratory of Data Analysis, Sobolev Institute of Mathematics, Siberian Branch of Russian Academy of Sciences, 630090 Novosibirsk, Russia; olga@math.nsc.ru

**Keywords:** type 1 diabetes, hypoglycemia, continuous glucose monitoring, machine learning, random forest, artificial neuron networks, prediction

## Abstract

Nocturnal hypoglycemia (NH) is a dangerous complication of insulin therapy that often goes undetected. In this study, we aimed to generate machine learning (ML)-based models for short-term NH prediction in hospitalized patients with type 1 diabetes (T1D). The models were trained on continuous glucose monitoring (CGM) data obtained from 406 adult patients admitted to a tertiary referral hospital. Eight CGM-derived metrics of glycemic control and glucose variability were included in the models. Combinations of CGM and clinical data (23 parameters) were also assessed. Random Forest (RF), Logistic Linear Regression with Lasso regularization, and Artificial Neuron Networks algorithms were applied. In our models, RF provided the best prediction accuracy with 15 min and 30 min prediction horizons. The addition of clinical parameters slightly improved the prediction accuracy of most models, whereas oversampling and undersampling procedures did not have significant effects. The areas under the curve of the best models based on CGM and clinical data with 15 min and 30 min prediction horizons were 0.97 and 0.942, respectively. Basal insulin dose, diabetes duration, proteinuria, and HbA1c were the most important clinical predictors of NH assessed by RF. In conclusion, ML is a promising approach to personalized prediction of NH in hospitalized patients with T1D.

## 1. Introduction

Nocturnal hypoglycemia (NH) is a wide-spread and potentially dangerous complication of insulin therapy which often goes undetected. In subjects with diabetes, almost 50% of all episodes of severe hypoglycemia occur at night. A growing body of evidence indicates that NH can cause sleep disturbances, morning headache, chronic fatigue, and mood changes; it is also associated with cardiac arrhythmias resulting in “death-in-bed syndrome” [1,2]. Hypoglycemia induces a wide range of changes in gene expression in the cardiovascular and nervous systems and may be a trigger for the damage of target organs [3]. Repeated episodes of hypoglycemia cause defective glucose counterregulation and contribute to the development of an impaired awareness of hypoglycemia [4].

Patients with type 1 diabetes (T1D) on basal bolus insulin therapy are particularly prone to NH [5]. In healthy subjects, hypoglycemia triggers awakening, but patients with T1D are often unable to wake up when their blood glucose drops [6]. Therefore, reliable and personalized predictive methods are urgently needed to reduce the risk of NH in T1D subjects. 

For a long time, the measurement of pre-bedtime glucose level was used for the NH risk assessment [7]. However, the value of the bedtime glucose in predicting NH is limited due to inter-individual and intra-individual differences in nocturnal glucose dynamics. A number of models based on clinical parameters, continuous glucose monitoring (CGM) data, indices of glycemic control, and glucose variability were proposed in recent years to identify patients at high risk of NH [8,9,10,11]. 

Machine learning (ML) technologies opened up new possibilities for personalized hypoglycemia forecasting. A comprehensive review [12] and meta-analysis [13] of research in this area were recently published. Currently, various ML algorithms have been tested for short-term NH prediction in subjects with T1D including Random Forest (RF) [14,15,16], Repeated Measures RF [17], Artificial Neural Networks (ANNs) [18], Support Vector Machine [14,19], Long Short-Term Memory [14], Linear Discriminant Analysis [9], and Multilayer Perceptron [19]. To be of practical use, a ML algorithm must provide enough time to take action to avoid hypoglycemia. In most of the above-mentioned studies, the prediction horizons (PHs) ranged from 15 to 60 min; in one study [15], it was extended to 6 h. 

Improving the predictive accuracy of ML models and assessing their applicability in various clinical situations remains an important challenge. The complimenting of glucose time series data with insulin doses, carbohydrate intake, and other clinical parameters, as well as combinations of different ML algorithms, is used to improve the predictive accuracy of the models [12]. In previous studies, ML algorithms were trained on CGM data obtained under normal living conditions. Another urgent task is the prediction of hypoglycemia in a hospital setting. It was demonstrated that in hospitalized patients hypoglycemia occurs with greater frequency between 0 and 6 a.m. [20]. Inpatient hypoglycemia in people with diabetes is associated with increased mortality and a longer hospital stay [21]. Previously, Fralik M et al. applied supervised ML for prediction of severe hypoglycemia in patients hospitalized under general internal medicine and cardiovascular surgery [22].

In this study, we aimed to develop ML-based models for short-term prediction of NH in hospitalized patients with T1D. We have also tested whether the inclusion of a broad set of clinical data and CGM-derived glucose variability parameters in the ML model, as well as the application of an oversampling or undersampling technique, can improve the accuracy of NH prediction. 

## 2. Materials and Methods

The process of ML model generation in our study included the following steps: (1) CGM data cleaning and preprocessing; (2) extracting metrics from CGM recordings; (3) data sampling; (4) combination of CGM data with clinical and laboratory parameters; (5) ML algorithm training; (6) evaluation of the model and NH predictors.

### 2.1. Databases

A database of CGM data obtained from 406 subjects with T1D was used to generate ML models for NH prediction. Data were collected from men and women aged from 18 to 70 years, on basal bolus insulin therapy. The treatment with sensor-augmented pumps with predictive low glucose suspend technology, current diabetic ketoacidosis or hyperglycemic hyperosmolar state, end-stage renal disease, congestive heart failure (class IV according to NYHA), malignant neoplasms, and acute infectious diseases were considered as exclusion criteria. Patients were observed at the clinic of RICEL—Branch of IC&G SB RAS, a tertiary referral hospital. All patients were admitted for a routine in-depth examination, screening for complications and correction of therapy. 

Blinded CGM was performed with an iPro™2, MMT-7741 (iPro2) CGM system and CareLink iPro™ (CareLink iPro, MMT-7340) software (Medtronic, Minneapolis, MN, USA). This system measures interstitial glucose values ranging from 2.2 to 22.2 mmol/L every 5 min. At least 4 capillary blood glucose measurements per day were performed with a One Touch Verio Pro+ glucose meter (Johnson & Johnson, New Brunswick, NJ, USA) to calibrate the CGM system. Mean CGM duration was 6.7 days; the range was from 3 to 11 days. 

The CGM database was matched to a clinical database containing demographic and anthropometric characteristics of the included subjects, information about diabetes, complications and associated diseases, data from laboratory tests, and instrumental examinations.

### 2.2. Model Building 

For the modeling, CGM records representing nocturnal intervals (from 00:00 to 05:59 a.m.) were used. The NH was defined as an episode of interstitial glucose level <3.9 mmol/L for at least 15 min [23]. 

#### 2.2.1. CGM Data Cleaning and Preprocessing

At the first step, we cleaned the data, looking for outliers and record defects. The CGM records with data gaps of 30 min or more were excluded. Shorter intervals of missed values were linearly extrapolated based on surrounding observations. At the preprocessing stage, we cut intervals of length T from the suitable CGM records and divided these intervals depending on the presence of an episode of NH at the selected PH value. Since the number of intervals without hypoglycemia (NH- intervals) was much higher than those with the episode (NH+ intervals) and their behavior for adjacent intervals looks quite similar, we considered a sample of NH- intervals with starting moments $t_{1},t_{1+s},t_{1+2s},...$ where s ≥ 1 is a gap parameter. The number of obtained intervals depended on T and s; for example, for T = 45 min and s = 4 we had 216 NH+ intervals and 36684 NH- ones.

#### 2.2.2. Extraction of CGM Metrics 

Since CGM data had a significant stochastic component and the amount of available data was not very large, feature-based procedures were used. Each record was represented as a series $\left\{G_{1},\ldots ,G_{n}\right\}$, where n = T/(5 min). From the appropriate sets of CGM records we derived parameters of glucose dynamics. These parameters included indices of glucose variability and glycemic control that are used in diabetology: coefficient of variation (CV), lability index (LI), low blood glucose index (LBGI), and 1 h continuous overlapping net glycemic action (CONGA-1) [24,25]. In addition, we applied indices used in the time series analysis: minimal value, difference between the last two values (DLV), acceleration over the last values (ALV), and linear trend coefficient (LC). Ultimately, 8 metrics were chosen (Table 1).

#### 2.2.3. Data Sampling 

As expected, the numbers of CGM intervals with a recorded NH episode were significantly less than that of the intervals without. To get a more balanced distribution of NH+ and NH- intervals in the training subset, we have applied oversampling and undersampling techniques. Oversampling consisted of perturbation with small Gaussian noise. For each feature, we used normal distribution N(0,σ), where parameter σ equals 5% of the standard deviation of the sample. This technique was applied for generating artificial CGM records with a NH episode. Undersampling consisted of selecting the most representative records without NH. To determine the representative records, we clustered NH- intervals using a k-medoids algorithm with a number of clusters equal to the number of NH events. The obtained medoids representing the intervals without NH were used for the consequent analysis. The effects of oversampling and undersampling techniques on the prediction accuracy were estimated. 

#### 2.2.4. Input Clinical Parameters into the Models 

At the next step, clinical characteristics of patients were entered into the models. In total, 23 clinical and laboratory parameters were assessed as potential contributors for NH risk. These parameters included age, sex, body mass index (BMI), diabetes duration, diabetic complications and associated diseases, insulin treatment characteristics, hypolipidemic and antihypertensive therapy, glycated hemoglobin A1c (HbA1c), renal function, and albuminuria (Table S1). 

#### 2.2.5. ML Algorithms

We conducted a number of preliminary experiments with different kinds of ML methods for constructing a prediction model. Finally, we decided to use RF, Logistic Linear Regression with Lasso regularization (LogRLasso), and ANN. RF is characterized by high generalization ability and robustness, especially in situations with redundant and possibly non-informative features [26]. LogRLasso is also a robust technique which provides an embedded opportunity to select the most important features [27]. The Levenberg–Marquardt algorithm, known for its fast convergence and robustness [28], was applied for ANN training. We used an ANN with a fully connected feed-forward network architecture with two hidden layers (5 neurons in each layer). 

### 2.3. Model Evaluation

The quality of prediction was evaluated using 10-fold cross-validation. The model parameters were evaluated for the PHs of 15 and 30 min.

If a decision taken by a classifier depended on a certain threshold, ROC curve analysis was performed. Assessment of the quality of classifiers was carried out by the estimation of area under the curve (AUC). This metric is independent of the decision threshold and can be used in situations of significant differences in class frequencies. In addition, the numbers of true positive, false positive, false negative, and true negative forecast results were calculated. Based on these parameters, sensitivity (Se) and specificity (Sp) of the models were estimated. 

### 2.4. Assessment of NH Predictors

We used RF as a standard tool for estimating the value of predictors in a model [26]. This method ranks all available features according to their usefulness in the prediction: the more frequently a feature is chosen in the ensemble of decision trees, and the more accurate predictions it yields, the higher the rank. There were 500 trees in the ensemble. In addition, we used LogRLasso to evaluate feature importance. Due to the embedded regularization, this method reveals non-significant features which are attributed with zero model coefficients. The method makes it possible to assess the direction of the influence of features on the outcome (in our case, whether the risk of NH increases or decreases with an increase in a feature value).

## 3. Results

### 3.1. Characteristics of Patients 

The clinical characteristics of patients are shown in Table 2. We observed individuals aged from 18 to 70 years (median 36 years), with diabetes duration 0.5–55 years (median 16 years). The HbA1c level was 8.1% (range: 4.7–15.1%). All patients were on basal bolus therapy with insulin analogues. One hundred and fourteen patients (28.1%) had severe hypoglycemia in their medical history. An impaired awareness of hypoglycemia, assessed by the Clarke method [29], was revealed in 148 (36.5%) subjects.

### 3.2. Evaluation of ML Models 

Three ML methods, including RF composed of 500 trees, LogRLasso, and ANN, were evaluated using baseline (no-sampling), oversampling, and undersampling procedures. We have also compared the models based on the CGM metrics only with those included combinations of CGM and clinical data (Table 3). 

The models based on the LogRlasso algorithm and operating only with CGM data were characterized by the highest AUC values (0.962 in a model with oversampling and 15 min PH; 0.932 in a model with oversampling and 30 min PH). At the same time, RF provided the best prediction accuracy when CGM and clinical data were combined (AUC: 0.97 in a model without sampling and 15 min PH; 0.942 in a model without sampling and 30 min PH). ANN provided slightly worse results in the models trained on CGM only and CGM and clinical data. 

The sampling effect was quite modest and depended on the ML algorithm and PH. In a one-way ANOVA, the effect of sampling on AUC was insignificant (*p* = 0.8 for all algorithms). An application of a no-sampling approach provided the highest AUC values in the RF model trained on the CGM and clinical data. 

### 3.3. Evaluation of NH Predictors 

Lower minimal glucose and LC, and higher LBGI, DLV, CONGA-1, proteinuria, basal insulin dose, diabetes duration, and HbA1c, as well as the presence of autonomic neuropathy, formed the list of the 10 most reliable NH predictors assessed by RF with a 15 min PH (Table 4). At a 30 min PH, lower minimal glucose and HbA1c, higher LBGI, DVL, daily and basal insulin doses, diabetes duration, proteinuria, eGFR, and BMI demonstrated the highest importance. Among the clinical factors, insulin dose, diabetes duration, and proteinuria were associated with the risk of hypoglycemia positively; meanwhile, HbA1c, eGFR, and BMI demonstrated negative associations. 

## 4. Discussion

The prevention of hypoglycemia, a frequent and potentially life-threatening complication of insulin therapy, remains a priority in diabetes care. Recent progress in the field is related to the implementation of sensor-augmented pumps with predictive low glucose suspend technology and closed-loop systems [30,31]. However, a significant proportion of patients with diabetes remain on multiple daily insulin injections. Therefore, it is important to develop reliable methods of hypoglycemia prediction for these patients also. In this study, we engineered ML models for real-time NH prediction in patients with T1D in a hospital setting. We assessed the predictive accuracy of the models based on CGM data and three ML algorithms: RF, LogRLasso, and ANN. We also evaluated the effectiveness of the use of clinical data as additional parameters, as well as oversampling and undersampling techniques, in the NH prediction.

In our models, RF provided the best prediction accuracy (in terms of AUC cross-validated estimates) at 15 min and 30 min PHs. LogRLasso was ranked as the second and ANN as the third algorithm. The more modest result of ANN can be explained by the relatively small sample size and the inherent stochastic nature of the data.

The choice of PH is an important step in the building of predictive models. In a recent review, Mujahid et al. indicated a 30 min PH as the most commonly used in ML-based models for NH prediction [12]. However, the optimal PH duration is still debatable, since the rate of development and severity of hypoglycemia, as well as the response to carbohydrates, can vary. Obviously, in the case of NH, the PH should not be too long; otherwise, the duration and quality of sleep can be reduced significantly. However, the PH should be long enough to enable patient or medical staff to take preventive actions. The American Diabetes Association advises patients to follow the “15:15” rule for the treatment of hypoglycemia: “have 15 g of carbohydrate to raise your blood sugar and check it after 15 min. If it’s still below 70 mg/dL, have another serving” [32]. Therefore, we believe that 15 min or 30 min PHs are acceptable in most cases.

An uneven distribution of observations between the classes, or the problem of imbalanced data, is a challenge in the building of ML models. In our sample, the number of CGM intervals with at least one episode of NH was much less than that of the intervals without an episode: depending on the PH, we have analyzed 209-256 intervals with NH and about 40,000 intervals without. In data analysis, oversampling and undersampling techniques are used to adjust the class distribution of a data set. These methods involve the generation of artificial observations of the minority class (oversampling, or augmentation technique) or the partial exclusion of observations from the majority class (undersampling) [33,34]. In this work, we have tried both oversampling and undersampling techniques and estimated the effects of these techniques on the prediction quality. The effect of the sampling depended on the ML method and the PH. The use of the oversampling provided slightly better results (in terms of AUC metric) compared to other techniques. At the same time, in the models generated by RF, the application of a no-sampling approach provided the highest quality of forecasting.

First, we trained ML models on CGM data only. The minimal glucose, LBGI, and DVL were the most reliable NH predictors at 15 min and 30 min PHs. Besides, CONGA-1 and LC were important in 15 min forecasting. At a 15 min PH, the highest AUC levels were 0.959% for RF, 0.962% for LogRLasso, and 0.947% for the ANN algorithm. In the models with a 30 min PH, the highest AUC values: 0.927%, 0.932%, and 0.924% were obtained by RF, LogRLasso, and ANN, respectively. Thus, parameters characterizing the concentration of glucose and the dynamics of glucose levels before the episode of hypoglycemia had the greatest prognostic value, as expected.

We have also investigated whether the inclusion of a set of clinical and laboratory data could improve the quality of CGM-based prediction. For this purpose, we input 23 parameters in the models, including demographic characteristics, information about diabetes, its complications and associated diseases, and laboratory test results. We did not include carbohydrate data, having taken into account frequent inconsistence of these data and the fact that most patients do not eat at night. Incorporating the clinical data in the models increased the sensitivity and specificity of the forecast up to 2% at a 30 min PH. Proteinuria, basal insulin dose, diabetes duration, and HbA1c turned out to be the most important clinical predictors of NH at 15 min and 30 min PHs. Besides, daily insulin dose, eGFR, and BMI were important for 30 min forecasting. 

In general, all the models we had built showed good prediction quality assessed by the sensitivity, specificity, and AUC. In particular, sensitivity and specificity varied from 94.5% and 91.4%, respectively, at a 15 min PH to 90.4% and 87.4% at a 30 min PH. 

Our study has some evident limitations. The duration of CGM was quite short. The datasets used were not very large and the number of observations with NH was limited. At the same time, as far as we know, this is the first study aimed to develop ML-based methods for short-term NH prediction in hospitalized patients with T1D. The resulting models can be used to develop a decision support system for the prevention of NH in hospitalized patients with T1D. 

## 5. Conclusions

In this study, we have developed a ML-based approach for predicting NH in patients with T1D in a hospital setting. The models trained on CGM data and operating RF, LogRLasso, and ANN algorithms showed acceptable prediction accuracy in terms of specificity, sensitivity, and AUC with PH lengths of 15 and 30 min. The incorporation of clinical data into the models improved the sensitivity and specificity of forecast up to 2%. Among the clinical parameters, basal insulin dose, diabetes duration, proteinuria, and HbA1c turned out to be the most reliable NH predictors. 

The development and implementation of decision support systems based on ML algorithms seems to be a promising approach to reduce the burden of NH in patients with T1D on multiple daily insulin injections.

## Figures and Tables

**Table 1:** CGM-derived metrics used for the engineering of ML models.

Parameter	Formula
CV	$CV=\frac{SD}{\bar{G}}\times 100\%$ $,\;\mathrm{where}\;\bar{G}=\frac{\sum_{i=1}^{n}G_{i}}{n}$ $\;\mathrm{and}\;SD=\sqrt{\frac{\sum_{i=1}^{n}\left(G_{i}-\bar{G}\right)}{n-1}}$
LI	$LI=\sum_{i=1}^{n-1}{\left(G_{i}-G_{i+1}\right)}^{2}/5$
LBGI	$LBGI=\frac{1}{n}\sum_{i=1}^{n}rl(G_{i})$, where $rl(G_{i})=r(G_{i})\;if\;f(G_{i})<0\;and\;0\;otherwise\;,\;$ $rh(G_{i})=r(G_{i})\;if\;f(G_{i})>0\;and\;0\;otherwise\;,\;$$r(G_{i})=10\times f^{2}(G_{i})$, $f(G_{i})=1.509\times \left[{\left(\log(18\times G_{i})\right)}^{1.084}-5.381\right]$
CONGA-1	$CONGA\left(1\right)=\sqrt{\frac{\sum_{i=2}^{n}\left(D_{i}-\bar{D}\right)}{n-1}}$ $\;\mathrm{where}\;\bar{D}=\frac{\sum_{i=2}^{n}D_{i}}{n-1}$ $,\;D_{i}=G_{i}-G_{i-1}$
Minimum value	$G_{\min}=\min\;(G_{1},\ldots ,G_{n})$
DLV	$G_{n-1}-G_{n}$
ALV	$\left(G_{n}-G_{n-1}\right)-\left(G_{n-1}-G_{n-2}\right)$
LC	$\mathrm{coefficient}\;b_{1}$ $\;\mathrm{in}\;a\;\mathrm{linear}\;\mathrm{trend}\;\mathrm{model}\;G_{i}=b_{0}+b_{1}\;t_{i}+\varepsilon_{i}$

Abbreviations: ALV, acceleration over the last values; CONGA-1, 1-h continuous overlapping net glycemic action; CV, coefficient of variation; DLV, difference between the last two values; LBGI, low blood glucose index; LC, linear trend coefficient; LI, lability index.

**Table 2:** Clinical characteristics of T1D patients.

General Demographic and Clinical Parameters	
Sex, m/f, *n* (%)	147/259 (36.2/63.8)
Age, years	36 (28–48)
BMI, kg/m^2^	23.6 (21.2–27.1)
Waist-to-hip ratio	0.84 (0.78–0.91)
Current smoking, *n* (%)	68 (16.7)
**Diabetes-related parameters and associated diseases**	
Diabetes duration, years	16 (10–25)
Daily insulin dose, IU	40 (29.1–53.6)
Daily insulin dose, IU/kg	0.59 (0.47–0.76)
Daily basal insulin dose, IU	19.0 (13.6–26)
Daily basal insulin dose, IU/kg	0.28 (0.21–0.38)
Diabetic retinopathy, *n* (%)	246 (60.6)
Chronic kidney disease, *n* (%)	274 (67.5)
Neuropathy, *n* (%)	301 (74.1)
Impaired awareness of hypoglycemia, *n* (%)	148 (36.5)
Arterial hypertension, *n* (%)	159 (39.2)
Coronary artery disease, *n* (%)	31 (7.6)
**Laboratory parameters**	
HbA1c, %	8.1 (7.1–9.2)
Total cholesterol, mmol/L	5.0 (4.2–5.9)
LDL cholesterol, mmol/L	3.0 (2.4–3.7)
HDL cholesterol, mmol/L	1.5 (1.3–1.7)
Triglycerides, mmol/L	1.0 (0.7–1.4)
Serum creatinine, µmol/L	81.9 (73.7–94.0)
eGFR, mL/min/1.73 m^2^	88.0 (73.0–100.0)
UACR, mg/mmoL	2.1 (2.0–7.65)

Continuous data are presented as medians (25th–75th percentiles). Abbreviations: BMI, body mass index; eGFR, estimated glomerular filtration rate; HbA1c, glycated hemoglobin A1c; HDL, high-density lipoprotein; LDL, low-density lipoprotein; T1D, type 1 diabetes; UACR, urinary albumin-to-creatinine ratio.

**Table 3:** Quality metrics (%) of the ML models for NH prediction.

PH	Sampling/Parameters		RF		LogRLasso		ANN	
			CGM	CGM + Clinical Data	CGM	CGM + Clinical Data	CGM	CGM + Clinical Data
**15 min**	**OS**	**Se** **Sp** **AUC**	93.6 (3.4) 90.1 (2.4) 0.958 (0.011)	90.9 (2.8) 91.8 (2.3) 0.953 (0.012)	93.6 (1.9) 91.9 (2.2) **0.962 (0.010)**	93.0 (3.0) 93.0 (2.0) **0.968 (0.014)**	90.5 (5.9) 91.4 (1.6) 0.946 (0.032)	90.8 (2.5) 89.1 (4.5) 0.935 (0.029)
	**NS**	**Se** **Sp** **AUC**	91.8 (1.2) 91.1 (3.9) **0.959 (0.020)**	94.5 (2.6) 91.4 (3.3) **0.97 (0.017)**	93.6 (3.4) 91.2 (2.5) 0.957 (0.021)	92.4 (2.5) 92.3 (3.7) 0.958 (0.025)	88.6 (3.6) 92.6 (3.1) 0.934 (0.032)	90.3 (3.1) 91.0 (1.6) 0.935 (0.027)
	**US**	**Se** **Sp** **AUC**	88.2 (5.2) 92.7 (2.1) 0.953 (0.023)	92.3 (3.4) 90.6 (1.3) 0.956 (0.009)	90.5 (6.7) 91.4 (1.4) 0.947 (0.036)	90.8 (4.7) 91.2 (2.4) 0.947 (0.018)	90.0 (4.7) 90.2 (2.8) **0.947 (0.033)**	91.9 (3.7) 88.9 (3.6) **0.945 (0.017)**
**30 min**	**OS**	**Se** **Sp** **AUC**	87.6 (1.9) 88.9 (3.1) **0.927 (0.03)**	86.6 (3.6) 87.0 (2.6) 0.911 (0.019)	90.4 (1.7) 87.5 (2.2) **0.932 (0.06)**	91.0 (3.5) 87.7 (3.7) 0.94 (0.012)	87.6 (3.9) 88.0 (4.0) 0.918 (0.031)	84.6 (5.2) 87.2 (5.5) 0.881 (0.034)
	**NS**	**Se** **Sp** **AUC**	87.1 (4.6) 87.1 (6.0) 0.92 (0.036)	90.4 (4.7) 87.4 (1.6) **0.942 (0.028)**	87.1 (4.0) 90.8 (1.9) 0.928 (0.012)	86.9 (4.0) 90.3 (1.9) **0.933 (0.012)**	86.6 (3.2) 88.7 (2.2) **0.924 (0.018)**	83.3 (4.2) 86.3 (2.8) 0.881 (0.049)
	**US**	**Se** **Sp** **AUC**	89.5 (3.6) 86.5 (2.8) 0.912 (0.031)	92.4 (3.1) 85.3 (1.2) 0.923 (0.021)	85.1 (5.6) 89.5 (1.8) 0.913 (0.027)	90.3 (3.2) 86.7 (1.9) 0.92 (0.03)	85.1 (5.3) 87.5 (2.7) 0.908 (0.028)	85.2 (3.6) 84.8 (2.2) **0.901 (0.023)**

The SD values of the estimates obtained with cross-validation process are shown in the parentheses. The highest AUC values for each PH and ML algorithm are highlighted in bold. Abbreviations: ANN, Artificial Neural Networks; AUC, area under the curve; CGM, continuous glucose monitoring; LogRLasso, Logistic Linear Regression with Lasso regularization; NP, nocturnal hypoglycemia; PH, prediction horizon; RF, Random Forest; OS, oversampling; NS, no sampling; US, undersampling; Se, sensitivity; Sp, specificity.

**Table 4:** The most important NH predictors revealed by RF in patients with T1D.

PH	Parameters	Importance	Effect
15 min	Minimal glucose	1.000	−
	LBGI	0.786	+
	DLV	0.723	+
	CONGA-1	0.625	+
	LC	0.542	−
	Proteinuria	0.494	+
	Basal insulin dose, IU/kg	0.488	+
	Diabetes duration	0.457	+
	Autonomic neuropathy	0.383	+
	HbA1c	0.379	−
30 min	Minimal glucose	1.000	−
	LBGI	0.845	+
	Daily insulin dose, IU/kg	0.770	+
	HbA1c	0.698	−
	Diabetes duration	0.693	+
	Basal insulin dose, IU/kg	0.666	+
	Proteinuria	0.653	+
	eGFR	0.652	+
	DLV	0.589	+
	BMI	0.577	−

Effect: the risk of NH increases as the parameter value increases (+); the risk of NH decreases as the parameter value increases (−). Abbreviations: BMI, body mass index; CONGA-1, 1 h continuous overlapping net glycemic action; DLV, difference between the last two values; eGFR, estimated glomerular filtration rate; HbA1c, glycated hemoglobin A1c; LBGI, Low Blood Glucose Index; LC, linear trend coefficient; NH, nocturnal hypoglycemia; RF, Random Forest; T1D, type 1 diabetes.

## Data Availability

The data supporting reported results are available in Supplementary Materials. The source data are available from the corresponding authors upon request.

## Supplementary Materials

The following supporting information can be downloaded at: https://www.mdpi.com/article/10.3390/jpm12081262/s1, Table S1: Clinical and laboratory parameters of T1D patients that were included in the models for NH prediction.

## Abbreviations

ANN	Artificial Neural Networks
AUC	area under the curve
AVL	acceleration over the last values
BMI	body mass index
CGM	continuous glucose monitoring
CONGA-1	1 h continuous overlapping net glycemic action
CV	coefficient of variation
DVL	difference between the last two values
eGFR	estimated glomerular filtration rate
HbA1c	glycated hemoglobin A1c
LBGI	Low Blood Glucose Index
LC	linear trend coefficient
LI	Lability Index
LogRLasso	Logistic Linear Regression with Lasso regularization
ML	machine learning
NH	nocturnal hypoglycemia
PH	prediction horizon
RF	Random Forest
Se	sensitivity
Sp	specificity
T1D	type 1 diabetes
t-SNE	t-distributed Stochastic Neighbor Embedding
UACR	urinary albumin-to-creatinine ratio
