# Supplementary material for: Machine Learning Models for Nocturnal Hypoglycemia Prediction in Hospitalized Patients with Type 1 Diabetes
Source: J Pers Med. 2022 Jul 31;12(8):1262. doi: 10.3390/jpm12081262 (PMC9409948; doi:10.3390/jpm12081262)
Supplement: Supplementary file 1 [file jpm-12-01262-s001.zip › jpm-1804098-supplementary.pdf]

Table S1. Clinical and laboratory parameters included in the ML models.

	Parameter	Units/Variant
1	Age	years
2	Sex	man/woman
3	BMI	kg/m2
4	Diabetes duration	years
5	Severe hypoglycemia in medical history	no/yes
6	Impaired awareness of hypoglycemia	no/yes
7	Target HbA1c	%
8	Insulin dose	$IU*kg^{-1}*day^{-1}$
9	Basal insulin dose	$IU*kg^{-1}*day^{-1}$
10	CKD, stage	C0-C4
11	Diabetic neuropathy, autonomic	no/yes
12	Dislipidemia	no/yes
13	Obesity	no/yes
14	Arterial hypertension	no/yes
15	Antihypertensive therapy	no/yes
16	Heart failure	no/yes
17	Heart failure, class	0-4
18	Current smoking	no/yes
19	HbA1c	%
20	eGFR	mL/min/1.73 m^2
21	Albuminuria	mg/day
22	UACR	mg/mmol
23	Proteinuria	mg/day

BMI, body mass index; CKD, chronic kidney disease; eGFR, estimated gliomerular filtration rate; HbA1c, glycated hemoglobin A1c; UACR, urinary albumin-to-creatinine ratio.

.
